# Effects of acupuncture treatment on postoperative gastrointestinal dysfunction in colorectal cancer: study protocol for randomized controlled trials

**DOI:** 10.1186/s13063-022-06003-7

**Published:** 2022-01-31

**Authors:** Xueyan Liu, Zhijie Wang, Hao Yao, Yanrong Yang, Huijuan Cao, Zhanhao Toh, Ruwen Zheng, Yi Ren

**Affiliations:** 1 Beijing University of Chinese Medicine, Beijing, China; 2 Department of Colorectal Surgery, Cancer Hospital Chinese Academy of Medical Sciences, Beijing, China; 3 Center for Evidence-Based Chinese Medicine, Beijing University of Chinese Medicine, Beijing, China; 4 Singa Care Medical, Singapore, Singapore; 5 Department of Acupuncture, Beijing University of Chinese Medicine Dong Fang Hospital, Beijing, China; 6 Department of Neurology and Stroke Center, Beijing University of Chinese Medicine Dongzhimen Hospital, Beijing, China

**Keywords:** Colorectal cancer, Postoperative, Acupuncture, Gastrointestinal function, Enhanced recovery after surgery

## Abstract

**Background:**

Postoperative gastrointestinal dysfunction (PGID) is a common complication arising from colorectal cancer surgery. Attributing factors, such as anesthesia, surgical retraction, and early intake of water, can inhibit gastrointestinal motility, causing constipation, reduction or absence of bowel sounds, nausea, vomiting, and other symptoms. Delayed recovery in gastrointestinal function can lead to intestinal obstructions or paralysis, anastomotic leaks, and other complications, affecting the patient’s recovery and quality of life negatively. Due to its complex pathophysiology, treatment for PGID in colorectal patients has remained a challenge. Acupuncture is an alternative therapy commonly used for postoperative recovery. This study aims to evaluate the therapeutic efficacy and safety of acupuncture on PGID. Through the complementation of acupuncture and enhanced recovery after surgery (ERAS) protocols, the advantages of acupuncture treatments could be demonstrated to promote its application in future clinical practice.

**Methods:**

The study design is a prospective randomized controlled trial (RCT). One hundred sixty postoperative colorectal cancer patients will be recruited from Cancer Hospital Chinese Academy of Medical Sciences (CICAMS). Subjects who fulfill inclusion criteria will be randomly assigned into the acupuncture group (AG) (*n* = 80) or control group (CG) (*n* = 80). AG will receive acupuncture treatment and perioperative care guided by ERAS protocols, and CG will only receive perioperative care guided by ERAS protocols. The intervention will begin on the first day post-surgery, continuing for 4 days, with a follow-up assessment in a month. Time of first postoperative flatus would be the primary outcome measure. Secondary outcome measures include the time of first postoperative defecation, time of first fluid intake, time of first ambulation, postoperative hospital stay, gastrointestinal reaction score, acupuncture sensation evaluation scale, laboratory tests, postoperative quality of life, readmission rate, and postoperative complications. All results are evaluated from baseline, post-treatment, and upon follow-up.

**Discussion:**

The results of the study would help elucidate evidence of the therapeutic effects of acupuncture on the recovery of postoperative gastrointestinal function. The objective of the study aims for the eventual inclusion of acupuncture in the ERAS protocol, allowing for wider application in clinical practice.

**Trial registration:**

ClinicalTrials.gov ChiCTR2000036351. Registered on August 22, 2020

## Administrative information

Note: the numbers in curly brackets in this protocol refer to SPIRIT checklist item numbers. The order of the items has been modified to group similar items (see http://www.equator-network.org/reporting-guidelines/spirit-2013-statement-defining-standard-protocol-items-for-clinical-trials/).

**Table:** 

Title {1}	Effects of Acupuncture Treatment on Postoperative Gastrointestinal Dysfunction in Colorectal Cancer: Study Protocol for Randomized Controlled Trials
Trial registration {2a and 2b}.	Clinicaltrials.gov:ChiCTR2000036351.[Registry ID: XYL] [registered on 22-08-2020] http://www.chictr.org.cn/showproj.aspx?proj=45184
Protocol version {3}	Version 2 of 26-10-2019
Funding {4}	This study is supported by the Outstanding Teacher Educational Research of Dong fang Hospital of Beijing University of traditional Chinese medicine, the 1166 Talent Training Program of Dong Fang Hospital, Beijing University of traditional Chinese Medicine and the Scientific Research Fund Project of Beijing University of traditional Chinese medicine.
Author details {5a}	XYL,HY,YRY. Beijing University of Chinese Medicine, Beijing, China. ZJW. Department of colorectal surgery, Cancer Hospital Chinese Academy of Medical Sciences, Beijing, China. HJC. Center for Evidence-Based Chinese Medicine, Beijing University of Chinese Medicine, Beijing China. ZHT. Singa Care Medical, Singapore, Singapore. RWZ. Department of acupuncture, Beijing University of Chinese Medicine Dong Fang Hospital, Beijing, China. YR. Department of neurology and stroke center, Beijing University of Chinese Medicine Dongzhimen Hospital, Beijing, China.
Name and contact information for the trial sponsor {5b}	Ruwen Zheng Department of acupuncture, Beijing University of Chinese Medicine Dong Fang Hospital, Beijing, China. zrw123@sina.com
Role of sponsor {5c}	RwZ all contributed to the design and management of the study protocol.

## Introduction

### Background and rationale {6a}

Colorectal cancer is a common malignant condition involving the gastrointestinal tract. With the aging population and changes in diet structures, incidence and mortality rates of colorectal cancer have seen a rising trend through the years [1]. Epidemiological studies have shown [2, 3] that in 2016, newly diagnosed cases and deaths of colorectal cancer in the USA were 135,000 and 50,000, respectively, and China has a higher ratio of newly diagnosed cases to death (50.8%) when compared with the USA (37%). In China, the incidence of colorectal cancer in males and females ranked 4th and 3rd, respectively, while mortality rates ranked 5th and 4th, respectively. The incidence of colorectal cancer for the age group below 50 has also shown an increasing trend. Surgical intervention remains the main treatment for colorectal cancer, despite surgical trauma and postoperative complications. Postoperative gastrointestinal dysfunction (PGID) is one of the most common complications and is often associated with administration of anesthesia, surgical retraction, postoperative fasting, usage of analgesics, prolonged bed rest, etc. [4, 5], and include symptoms such as distention and absence of flatus or bowel, accompanied by varying degrees of nausea, vomiting, and fever, leading to longer recovery periods, decrease in quality of life, and increase consumption of medical resources [6]. Current methods to promote recovery of gastrointestinal function include postoperative conventional treatments and prokinetic drugs such as domperidone and cisapride. However, the therapeutic efficacy of these drugs is less than ideal and accompanied with various side effects, possibly due to over-stimulation of gastrointestinal motility that could affect postoperative recovery negatively [7].

Enhanced recovery after surgery (ERAS) is an evidence-based approach that employs a series of multimodal and optimized perioperation measures to reduce surgical trauma and stress response, promote physiological and psychological well-being, and facilitate faster recovery [8]. As a newly implemented perioperative management principle, specific measures include preoperative education and counseling, selective preoperative bowel preparation, selective preoperative sedation, 30–60-min preoperative antibiotic prophylaxis, standardized anesthetic protocol, perioperative fluid intake management, prevention of intraoperative hypothermal, early removal of urinary catheters, multimodal postoperative analgesia, early postoperative food intake, early mobilization, etc. [9]. ERAS protocol has the advantages of faster recovery, lesser complications, shorter hospitalization, and lower medical cost and is widely adopted and implemented in various surgical fields [10]. With rapid developments in the field of surgical rehabilitation, measures to improve postoperative recovery in colorectal cancer patients are gradually being optimized. At present, although ERAS has lowered the incidence of postoperative complications, a portion of patients, in particular elderly, diabetic patients, or individuals who have poor digestive functions, could still develop long-term, refractory, recurrent complications [11].

Acupuncture is a unique and natural treatment method originating from China, and the practice of which to treat digestive ailments had been recorded over a few thousand years [12]. Recent evidence shows that [13, 14] acupuncture exhibits higher effectiveness over western medications, displaying various advantages such as expediting the recovery of gastrointestinal function, reducing postoperative complications, boosting immunity, and improving quality of life, when applied as a complementary treatment for colorectal cancer. Additionally, acupuncture possesses qualities of a safe and economical treatment and holds an irreplaceable role in rehabilitation medicine [15]. However, existing studies have their limitations, including small study samples, poorly designed experiments, low credibility of experimental results, etc. Our study aims to further investigate the therapeutic efficacy of acupuncture on postoperative recovery of gastrointestinal functions, by comparing trial results through complementation of acupuncture and ERAS protocol-guided laparoscopic radical resection of colon cancer versus solely ERAS protocol-guided laparoscopic radical resection of colon cancer, to provide statistical evidence for the inclusion of acupuncture in the ERAS protocols for colorectal cancer.

### Objectives {7}

1. To determine the therapeutic efficacy of acupuncture on the recovery of postoperative gastrointestinal function in colorectal cancer patients
2. To provide statistical evidence for the inclusion of acupuncture into ERAS protocols

### Trial design {8}

**Fig. 1:**
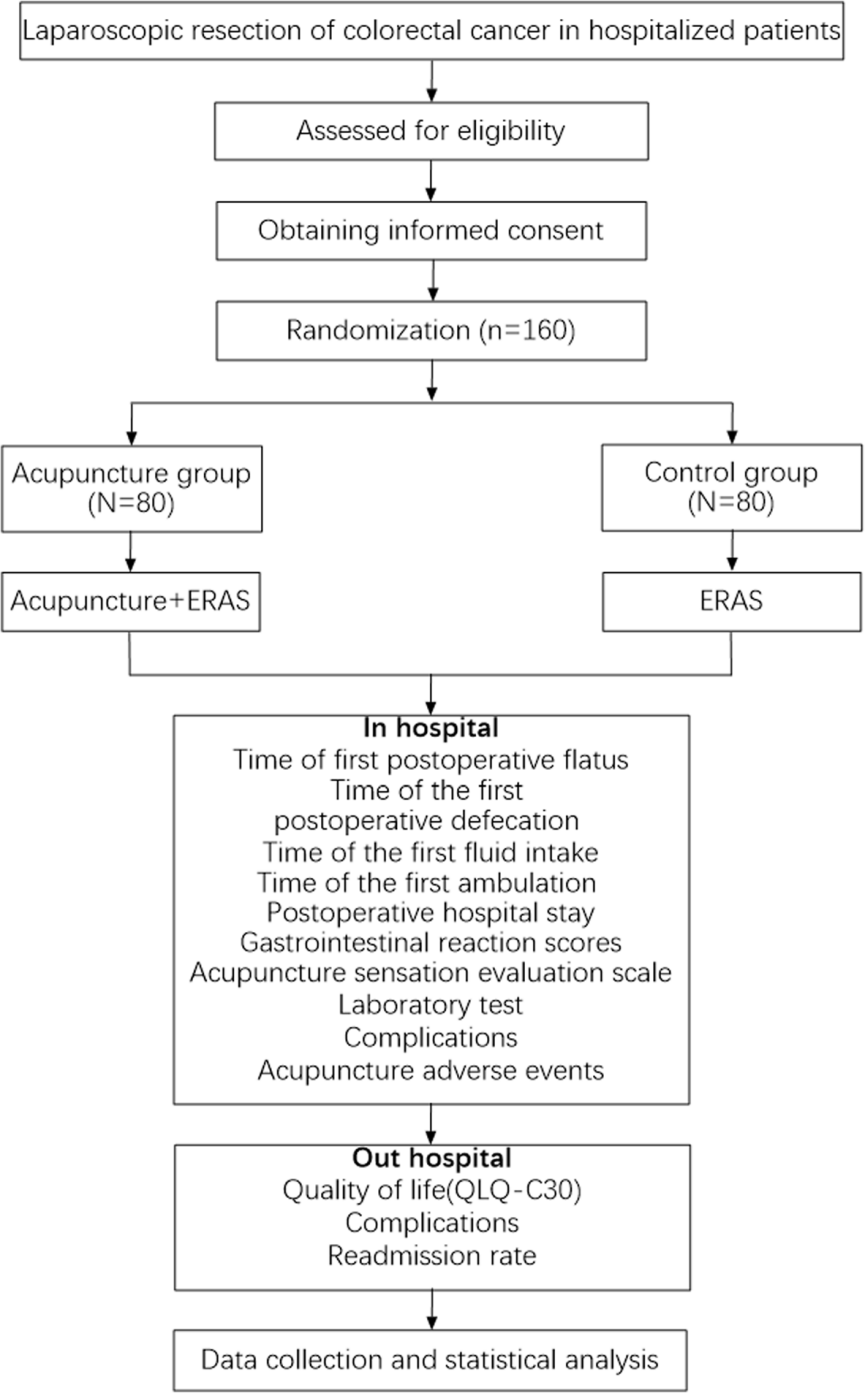
Flowchart of the study design

The study is a single-center, prospective, parallel, randomized controlled clinical trial. Subjects who fulfill inclusion criteria and signed the informed consent form will be randomly assigned to either the acupuncture group (AG) or control group (CG) in a 1:1 ratio. Assessments are made prior to treatments and on the 4th day and 1 month after the treatments (Fig. 1).

The purpose of this study is to design a high-quality randomized controlled trial and realize the complete randomization of participants, and the evaluators and data analysts are blinded, so as to provide reliable data for the recovery of gastrointestinal function of colorectal cancer after acupuncture treatment. However, the framework of this trial is not perfect. First, this study failed to establish false acupuncture, and the comforting effect of acupuncture cannot be ruled out. Second, the included cases are completely random without too many restrictions, but the clinical situation of patients is complex, and the tumor stage, operation mode, and TCM syndrome type may be related to the speed of postoperative gastrointestinal function recovery. It is better to carry out stratified observation. Third, both patients received perioperative care guided by eras, which achieved the ceiling effect, and the acupuncture effect may not be obvious. Fourth, the patients knew the grouping situation and were not blinded.

## Methods: participants, interventions, and outcomes

### Study setting {9}

Patients who are scheduled to undergo laparoscopic radical resection for colorectal cancer will be recruited from the Colorectal Surgical In-patient Department of Cancer Hospital Chinese Academy of Medical Sciences (CICAMS). Patients are considered for inclusion if they meet the criteria as defined below.

### Eligibility criteria {10}

#### Inclusion criteria

To enroll in the study, subjects must (1) be diagnosed with colorectal cancer based on cytological or histopathological features, (2) be scheduled to undergo laparoscopic radical resection for colorectal cancer, (3) age between 18 and 80 years old, (4) consent to participation and completion of the full treatment process and be able to sign the informed consent form, and (5) postoperative blood examination showed no obvious abnormality.

#### Exclusion criteria

Patients may not participate in the study if they are as follows: (1) patients converted to open surgery; (2) have emergency such as acute intestinal obstruction or intestinal perforation; (3) have a history of other malignant neoplasms or are having other malignant tumors concurrently; (4) have undergone vagotomy or colostomy; (5) have preexisting conditions such as severe cardiovascular disease, hepatic, renal, hematopoietic, immune system conditions, and psychiatric disorders; (6) have skin allergies or infections; (7) are anxious or fearful of acupuncture treatments; (8) have been prescribed adrenergic agonist or antagonist, cholinergic, or anticholinergic medications that affect gastrointestinal function for the past 1 month; (9) have severe postoperative complications that require admission to the intensive care unit; (10) are pregnant or lactating; and (11) have American Society of Anesthesiologists (ASA) physical status classification IV.

### Who will take informed consent? {26a}

Based on the above criteria, to confirm whether a patient undergoing colorectal cancer surgery is eligible to participate in this study after screening, the patient has been assessed as qualified by the treatment group, and the surgeon will fill in the preliminary study information of the patient. Patients were invited to meet with researchers and doctors to discuss any remaining issues and sign informed consent.

### Additional consent provisions for collection and use of participant data and biological specimens {26b}

In this study, biological samples of participants will be collected as acupuncture safety indicators, which will be used as additional terms when participants sign the informed consent form, inform patients of the purpose of biological samples and possible privacy risks, and obtain the consent of participants.

## Interventions

### Explanation for the choice of comparators {6b}

The control group received routine postoperative ERAS protocol. The control group can take medicine according to the doctor’s advice.

### Intervention description {11a}

Study subjects from both groups will receive standardized perioperative care from ERAS protocols (detailed nursing and care plans will be facilitated by the CICAMS and would not be intervened by investigators) (Table 1).

**Table 1:**
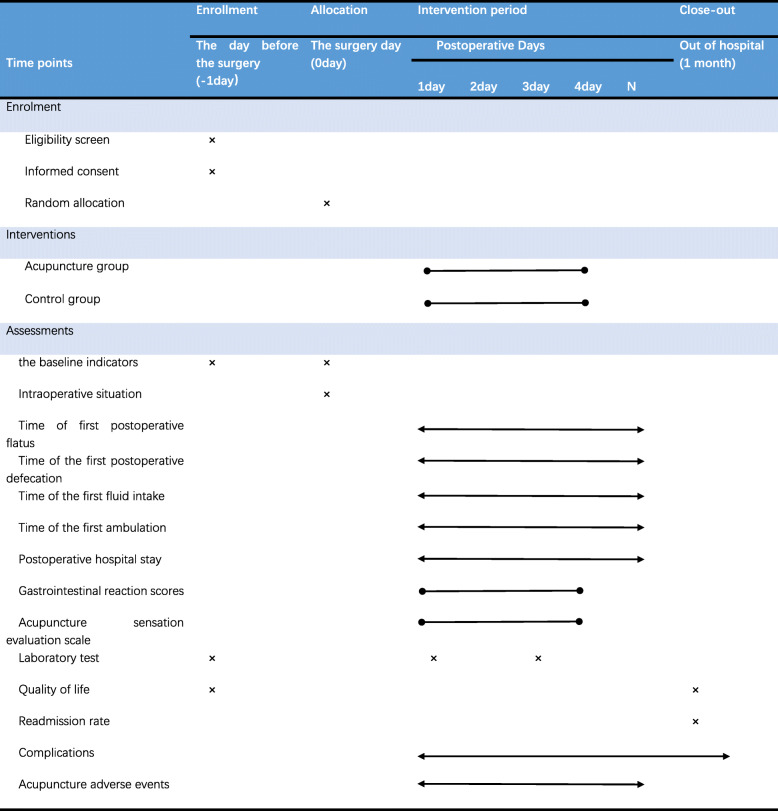
Time schedule of enrollment, interventions, and assessments

#### Perioperative care guided by ERAS protocols

1. Preoperative education, unconventional preoperative mechanical bowel preparation

2. Standardize anesthesia scheme, intraoperative warming

3. Comprehensive postoperative analgesia, remove the urinary catheter as soon as possible, early postoperative oral feeding, and early postoperative ambulation

### Acupuncture group

**Fig. 2:**
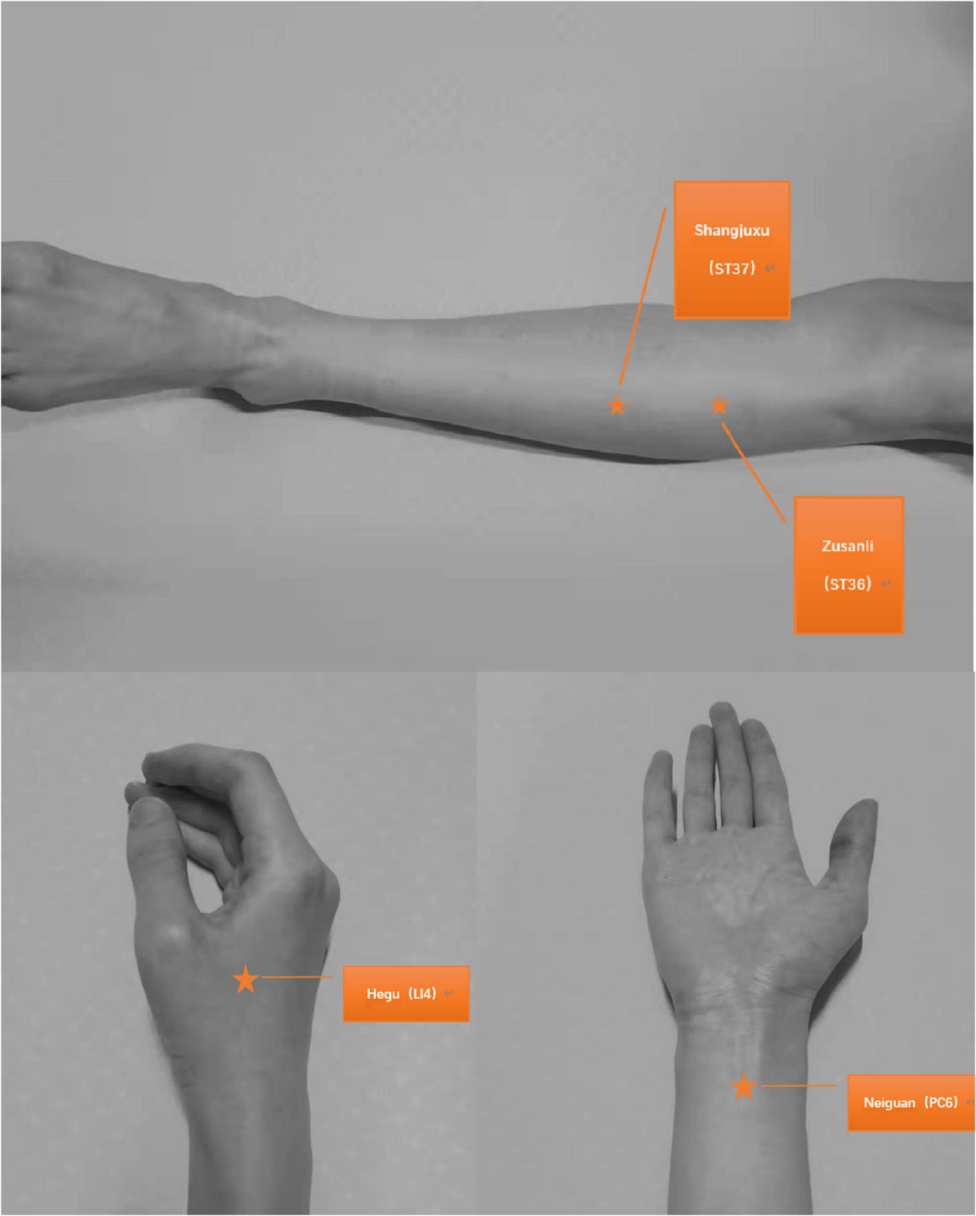
The location of ST36, ST37, L14, and PC6. ST36 is the Zusanli acupoint; ST37 is the Shangjvxu acupoint; L14 is the Hegu acupoint; PC6 is the Neiguan acupoint

Patients in AG will receive acupuncture treatment in addition to perioperative care guided by ERAS protocols. Acupuncture treatment will be performed once daily, consecutively over 4 days, starting on the first day after the laparoscopy. Patients will assume a supine position for the treatment. Treatment will be carried out by a licensed acupuncturist who holds a China Acupuncturist Certification, with at least 2 years of clinical experience in acupuncture. The site of insertions will be swabbed and disinfected with 75% alcohol prior to needle insertion. Sterile, single-use acupuncture needles with diameter 0.30 mm and length 40 mm (China Suzhou, Huatuo) are used. Acupuncture points are selected based on Traditional Chinese Medicine (TCM) theory and clinical experience: ST36 Zusanli, ST37 Shangjuxu, PC6 Neiguan, LI4 Hegu (Fig. 2). Exact locations and depth of insertions are referenced from the 2006 National Standards of the People’s Republic of China (GB/T12346-2006) “Nomenclature and Location of Acupuncture Points” [16] (Table 2). After insertion of needles, stimulation of acupoints will be performed every 10 min, and each operation lasts at least 10 s, through lifting-thrusting and twirling-twisting manipulations, to achieve sensations of *deqi*. Needles are kept inserted for 30min.

**Table 2:** Locations and manipulations of the acupuncture group and control group

Group	Acupoints	Locations	Needle insertion	Needle manipulation
Acupuncture group	Neiguan (PC-6)	On the palmar side of the forearm, 2 inches above the transverse line of the wrist, between the palmaris longus tendon and the flexor carpi radialis tendon	Perpendicular needle insertion at depth of 10–30 mm	Manipulation and De-qi sensation felt by practitioner and patient
	Hegu (LI-4)	On the back of the hand, between the first and second metacarpal bones, at the midpoint of the radial side of the second metacarpal bone	Straight needle insertion at depth of 10–30 mm	
	Zusanli (ST-36)	On the anterolateral side of the calf, 3 inches below the nose, a transverse finger (middle finger) from the front edge of the tibia	Straight needle insertion at depth of 30–60 mm	
	Shangjixu (ST-37)	On the anterolateral side of the calf, 6 inches below the nose, a transverse finger (middle finger) from the front edge of the tibia	Straight needle insertion at depth of 30–60 mm	
Control group	Non-intervention			

### Control group

Patients in CG will only receive ERAS care with no acupuncture intervention.

### Criteria for discontinuing or modifying allocated interventions {11b}

Patients can leave the study at any time for any reason if they wish to do so without any consequences. The patient’s participation in this study can also be ended by the investigator if the patient is uncooperative and/or does not attend study visits. The patient data that have been collected up to that moment will be included in the analysis. In case too many data are missing, the patient will be replaced by a new patient. If there are adverse events or postoperative complications, the study will be terminated early. In case of illness, patients will be asked to contact the primary investigator. Patients that are discovered after surgery not to meet the criteria will not receive acupuncture treatment and will be treated according to the standard of care. These patients will be removed from the study. Patient data included up to that moment will be included in the analysis.

Criteria for study termination include the following: ① The pain of acupuncture was intolerable or serious adverse reactions occurred; ② during the treatment, the symptoms worsened or other critical diseases appeared, and the patients could not continue the treatment; ③ researchers found that there were serious safety problems; ④ because of various reasons cannot adhere to the treatment.

### Strategies to improve adherence to interventions {11c}

The acupuncture treatment scheme is safe and can be adjusted according to the needs of patients, so the compliance rate of the protocol is very high, and the whole process of acupuncture treatment will be supervised by professional acupuncturists in our center.

### Relevant concomitant care permitted or prohibited during the trial {11d}

During the trial, other drugs affecting gastrointestinal function can be used according to the doctor’s advice. If used, detailed records are required. If patients have intolerable postoperative pain or other postoperative symptoms, conventional drugs can be used for emergency treatment.

### Provisions for post-trial care {30}

After waiting for the end of the experiment, the control group will receive 4 acupuncture treatments free of charge. If the subject is injured by acupuncture during the study period, acupuncture should be stopped immediately, symptomatic treatment should be given free of charge, and psychological counseling should be given to the patient at the same time. If the injury is serious, invite relevant experts for treatment and compensate for medical expenses.

### Outcomes {12}

#### Primary outcome

Time of first postoperative flatus is the study’s primary outcome and is measured as the time period starting from the patient’s return to ward after the laparoscopy procedure till the first passage of flatus. It is obtained through the assistance of family members and caregivers, via inquiry and record of patient’s status.

#### Secondary outcomes

Secondary outcomes include the time of first postoperative defecation, time of first fluid intake, and time of first ambulation, which are obtained through the assistance of family members and caregivers, via inquiry and records of patient’s status. Other secondary outcome measures such as gastrointestinal reaction score (postoperative pains, distention, nausea, vomiting), acupuncture sensation evaluation scale, laboratory test, postoperative hospital stay, quality of life, readmission rate, and other postoperative complications (fever, pneumonia, wound infection, bleeding, etc.) are assessed and evaluated by our investigators. The first postoperative evaluation of all patients was performed 12–24 h after operation to ensure that the patients were awake.

For gastrointestinal reaction score, postoperative pain will be evaluated regularly at three times a day using the visual analog scale (VAS), and the three scores will be averaged and tabulated with dosage and frequency of patient-controlled analgesia (PCA) and analgesic medication used. The extent of distention is recorded using the Likert scale [17], postoperatively from day 1 to 4. The extent of nausea is recorded using the VAS, postoperative from day 1 to 4. Vomiting will be counted daily.

For Acupuncture Sensation Evaluation Scale, acupuncture sensations are assessed after every acupuncture treatment session through inquiring about patient’s sensations.

For laboratory test, venous blood will also be collected on the day prior to operation, as well as the first and third day after the laparoscopy, to assess the safety of acupuncture base on blood examinations and biochemical indicators.

Postoperative hospital stay is measured from the day of the laparoscopy procedure till the day of discharge.

Quality of life is assessed through the Quality of Life Questionnaire (QLQ-c30), provided to patients prior to operation and 1 month after the operation [18].

For readmission rate, evaluate the readmission of patients 1 month after the operation, record the reasons for readmission in detail, and finally calculate and analyze.

Adverse events include postoperative complications and adverse acupuncture events. Postoperative complications are recorded and evaluated using the Clavien Dindo Classification [19]. Adverse acupuncture events including subcutaneous hematoma, continuous acupuncture afterfeeling, and needle stagnation will be recorded by the assessor.

### Participant timeline {13}

The participant timeline is presented in Table 1.

### Sample size {14}

Our study estimates that a mean decrease of 8 h in time of first flatus after application of acupuncture to promote recovery in postoperative gastrointestinal functions is of significant value. The literature review showed the standard deviation of time of first flatus between the study group and control group to be 12.28 and 14.57, respectively. Using a unilateral test of superiority, PASS.11 software is used to compute sample size. Using *α* = 0.05, *β* = 0.1, and power = 0.9, 68 subjects are required in each group, with a total of 136 subjects needed for the study. Estimating a 20% dropout rate, a minimum recruitment of 160 subjects is required.

### Recruitment {15}

One hundred sixty patients are recruited from the Colorectal Surgical In-patient Department of CICMAS through publication and distribution of posters and leaflets containing information of the study.

### Assignment of interventions: allocation

#### Sequence generation {16a}

Randomized allocation sequence will be generated using Statistical Analysis System, SAS 9.3 (SAS Institute Inc. Cary, NC, USA), by an appointed investigator.

#### Concealment mechanism {16b}

The generated sequences will be placed in serialized, opaque, sealed envelopes (to ensure concealment of allocation from the evaluators).

#### Implementation {16c}

After ensuring patients fulfill inclusion criteria and informed consent forms are obtained from them, the envelopes are opened by the acupuncturist and acupuncture treatment is carried out (the acupuncturist is excluded from assessment and statistical analytic process as blinding of the acupuncturist cannot be achieved). The study group will be revealed at the same time to both the patient and researcher.

### Assignment of interventions: blinding

#### Who will be blinded {17a}

Throughout the study, the statisticians and evaluators are blinded, so that independent assessments and statistical analysis can be carried out. The subjects in the control group could not be blinded due to their particularity. Acupuncturists were not blinded in this study.

#### Procedure for unblinding if needed {17b}

Unblinding will only be allowed under emergencies such as the incidence of serious adverse effects.

### Data collection and management

#### Plans for assessment and collection of outcomes {18a}

The data will come from the electronic medical record (EMR) and will be collected using the case report form (CRF). A questionnaire survey was conducted by doctors. Laboratory tests are performed by the laboratory and all data obtained during the study will be anonymized and stored in the study folder on our protected study server. Only the research team has access to this particular research folder. The operation time, intraoperative rehydration, the use of anesthetics, the number of lymph nodes removed, and the length of hospital stay will be obtained from the EMR. In addition, the results of the quality of life scale (QLQ-C30) will be used to evaluate the postoperative quality of life.

#### Plans to promote participant retention and complete follow-up {18b}

The patients will receive extensive information about the study set-up and requirements during the recruitment. The importance of completion of the follow-up will be stressed. Patients are allowed to stop acupuncture at any time during the study and are not obliged to give a reason to discontinue. If possible, the patient will be asked to complete the survey at 1 month after acupuncture treatment. All patients are reminded throughout the study to fill out the questionnaires during study visits. Throughout the follow-up period, the researchers will check responses and, if necessary, contact patients for completion of their follow-up.

#### Data management {19}

Patient data will be collected through ECRF. QLQ-C30 will be filled in by telephone and will be stored in SPSS. Back-ups in the study folder on the protected research server will be made regularly (once per 1 month). Informed consent and end-of-trial dates will be recorded in the electronic patient dossier, and signed paper forms will be stored within our hospital in a locked room. (S)AE(I)s will be recorded in the eCRF. To be able to reproduce study results and to help future users to understand and reuse data, all changes made to the raw data and all steps taken in the analysis will be documented in the eCRF and IBM SPSS. Source data will remain available in the electronic patient record. All research data, including patient material, will be archived for 30 years after the study has ended.

#### Confidentiality {27}

Research data will be stored using a study identification code for each participant. The key to the identification code list will only be available to the research team during the study and will be documented and safeguarded by the principal investigator according to research guidelines after completion of the study. No patient identification details will be reported in publications.

Plans for collection, laboratory evaluation, and storage of biological specimens for genetic or molecular analysis in this trial/future use {33}

Not applicable. The biological samples collected in this study are only used as safety evaluation indicators and will not be used for genetic or molecular analysis. The biological samples will be destroyed immediately after monitoring, which will not infringe on the rights and interests of participants, let alone violate ethics.

### Statistical methods

#### Statistical methods for primary and secondary outcomes {20a}

Spss22.0 software was used to analyze and process the clinical observation data, in which in the descriptive statistical analysis, qualitative indicators were described by frequency table and percentage and quantitative indicators were described by mean and standard deviation. Comparison of the two groups was performed: quantitative data in line with normal distribution using the *t* test, homogeneity test of variance between groups, with 0.05 as the test level, uneven variance using Satterthwaite method for correction of *t* test, and not in line with normal distribution using Wilcoxon rank-sum test. The hypothesis test uses a two-sided test and gives the test statistics and the corresponding *p* value, with *p* ≤ 0.05 as statistically significant. All (S)AE(I)s will be summarized and recorded including the nature, date and time of onset, date of resolution, determination of seriousness, severity, action taken, outcome, and possible causality to study treatment. SAE data will be presented in a descriptive manner.

#### Interim analyses {21b}

There are no interim analyses planned.

#### Methods for additional analyses (e.g., subgroup analyses) {20b}

There are no subgroup analyses planned.

#### Methods in analysis to handle protocol non-adherence and any statistical methods to handle missing data {20c}

The primary outcome will be assessed using an intention-to-treat analysis. Missing data will be reduced to a minimum by using the appropriate measures described above. Mixed models do not require imputations for missing data. If any statistical method is needed to account for missing data in the secondary outcomes, multiple imputation will be used.

#### Plans to give access to the full protocol, participant-level data, and statistical code {31c}

The datasets used and/or analysed during the current study can be made available by the corresponding author upon reasonable request and in agreement with the research collaboration and data transfer guidelines of the Dong Fang Hospital, Beijing University of Chinese Medicine.

### Oversight and monitoring

#### Composition of the coordinating center and trial steering committee {5d}

This is a single-center study designed in Dong Fang Hospital of Beijing University of traditional Chinese medicine and implemented and coordinated in Cancer Hospital of Chinese Academy of Medical Sciences.

Daily support for the trial was provided by:

Main responsible person: responsible for supervising the trial and the medical responsibility of patients.

Data manager: organize data capture and protect quality and data.

Study coordinator: trial registration, informing patients, coordinating study visits, and annual safety report.

Research surgeons: recruit patients, evaluate their postoperative recovery, and ensure follow-up in accordance with the agreement.

The study team meets biweekly. There is no trial steering committee or stakeholder and public involvement group.

#### Composition of the data monitoring committee, its role, and reporting structure {21a}

No data security monitoring board (DSMB) was designated in this study. The decision was based on the absence of serious adverse events in the pre-trial. In addition, since this is not a double-blind trial, there is no need for DMSB to protect researchers and doctors from blindness. A colorectal cancer surgeon will be designated as the safety officer for this study. In case of serious adverse events, the safety officer will be contacted within 48 h. The safety officer will assess whether the serious adverse event is (definitely or possibly) related to the treatment. If there is a (possible) handling relationship, further safety measures will be taken based on the advice of the safety officer.

#### Adverse event reporting and harms {22}

All adverse events reported by the subject or observed by the investigators will be recorded. The causality to the study treatment event will be recorded. Some complications are considered as adverse events of special interest (AESI): such as fever, bleeding, wound infection, intestinal obstruction, gastroenteric paralysis, anastomotic leakage, pneumonia, deep vein thrombosis, etc. Serious adverse events (SAEs) should be reported to the ethics committee of Dong Fang Hospital in time.

Safety evaluation of acupuncture: needle breaking, needle leaving, needle fainting, local hematoma, infection, and abscess; incidence and average times of other discomfort after acupuncture (pain, nausea, vomiting, palpitation, dizziness, headache, anorexia, and insomnia). Form a specific record form, and ask whether the above situation occurs during the diagnosis and treatment of each patient. If so, record it immediately.

#### Frequency and plans for auditing trial conduct {23}

A research supervisor was appointed according to the guidelines for a special research audit of Dong Fang Hospital of Beijing University of traditional Chinese medicine. The supervisor conducts an annual visit to check the existence and integrity of the investigation documents. In addition, 25% of patients were randomly selected for the following data: informed consent, inclusion and exclusion criteria, source data, and missing and reporting of AEs. For more information, refer to the monitoring plan.

#### Plans for communicating important protocol amendments to relevant parties (e.g., trial participants, ethical committees) {25}

All substantial amendments to the study will be notified to the ethics committee and the competent authorities of the Dong Fang Hospital of Beijing University of Chinese medicine. Non-substantial amendments will be recorded and filed. In case amendments concern or affect participants in any way, they are informed about the changes. If needed, additional consent will be requested and registered. Also, online trial registries will be updated accordingly.

### Dissemination plans {31a}

Results of this research will be disclosed completely in international peer-reviewed journals. Both positive and negative results will be reported.

## Discussion

Gastrointestinal dysfunction is a major factor that affects postoperative recovery in colorectal cancer patients and could lead to poor prognosis. Therefore, prevention of PGID in colorectal cancer patients to reduce perioperative complications and hospitalization cost is of important significance [20]. Current western medical interventions such as gastrointestinal decompression and oral administered prokinetic medications have shown unsatisfactory results in patients, leading to the search for alternative treatment options by clinical practitioners, to facilitate early recovery of gastrointestinal functions [7]. Acupuncture is a safe and effective treatment method that could promote gastrointestinal functions while having minimal side effects [13, 15]. Although current studies have shown the duration of PGID could be reduced through acupuncture, research on acupuncture and its role and therapeutic effect in the prevention of PGID in colorectal cancer patients has not been studied [21]. With recent developments in enhanced recovery principles, improving postoperative quality of life in patients has gained importance [22]. Therefore, our study aims to determine the effects of acupuncture intervention protocols on the recovery of postoperative gastrointestinal functions in colorectal cancer patients.

Postoperative gastrointestinal dysfunction in colorectal cancer patients is recognized as “Chang Bi” or “Chang Jie” in traditional Chinese medicine theory. Based on TCM organ system theory, acupoints are mostly selected from the spleen and stomach meridian to promote recovery of gastrointestinal functions. Acupoints include LI 4, PC6, ST36, and ST37. ST36 improves blood flow and distribution within the gastrointestinal tract, regulates motilin production through somatostatin, and improves peristalsis through regulation of NO which increases the production of angiotensin [23, 24]. ST37 is a lower confluent acupoint for the large intestines and is often used in conjunction with ST36 to treat large intestine-related conditions such as ulcerative colitis and irritable bowel syndrome [25]. PC6 regulates endocrine functions, in particular epinephrine and vasopressin, to reduce stomach acid secretions and regulate gastrointestinal functions [26]. PC6 is also frequently used in the prevention of postoperative nausea caused by the use of opioids and other medications [27]. LI4 is the yuan-primary point of the large intestine meridian and is often used in the treatment of large intestine-related conditions and for its analgesic properties [28].

Patients between ages 18 and 80 have been included in the study to cover a larger spectrum of the demographics. At the same time, patients after colorectal cancer surgery were completely randomized, and the outcome evaluators and statisticians were blinded, and the reliability of the trials was high. In addition, assessment of gastrointestinal function-related clinical parameters and laboratory tests is done to aid in the evaluation of the efficacy and safety of acupuncture treatments, ultimately providing the basis for the overall evaluation of the effects of acupuncture on recovery of gastrointestinal function from colorectal cancer operations. Clinical parameters for gastrointestinal function include the time of first postoperative flatus, time of first postoperative defecation, time of first fluid intake, hospitalization period, etc. Importantly, laboratory tests such as blood test and biochemical indicators have been included in our study to assess the safety of acupuncture. One of the advantages of our study is the conduct of a follow-up to gather response through the QLQ-c30 to assess patients’ quality of life 1 month after the operation, which will reflect the long-term impact of acupuncture on postoperative colorectal patients.

However, there are limitations to the study. Firstly, ensuring punctual and accurate follow-ups is both an important and difficult task, and insertion of rectal tubes in a portion of our study subjects might cause inaccuracies in reporting the time of first postoperative flatus by the patients. Secondly, the study lacks a sham acupuncture control group, due to difficulties in blinding of study subjects. Lastly, both groups received perioperative nursing guided by ERAS protocol, so the role of acupuncture may be ignored. Despite these limitations, the study should elucidate the general effect of acupuncture on postoperative recovery.

In conclusion, we hope that through rigorous clinical study, statistical evidence of acupuncture in promoting recovery of gastrointestinal function in post-laparoscopy colorectal cancer patients can be demonstrated, providing the basis for the inclusion of acupuncture into ERAS principles and protocols, to encourage the application of acupuncture in the aforementioned condition after conventional surgical procedures.

## Trial status

Recruitment began on 16 October 2020. Recruitment is anticipated to end in September 2022. Recruitment for the study is currently ongoing.

## Abbreviations

PGID: Postoperative gastrointestinal dysfunction
ERAS: Enhanced recovery after surgery
RCT: Random controlled trial
CICAMS: Cancer Hospital Chinese Academy of Medical Sciences
AG: Acupuncture group
CG: Control group
ST36: Zusanli
ST37: Shangjuxu
PC6: Neiguan
LI4: Hegu
CRF: Case report form
QLQ-c30: Quality of Life Questionnaire
